# Supplementary material for: Proportion of Respiratory Syncytial Virus, SARS-CoV-2, Influenza A/B, and Adenovirus Cases via Rapid Tests in the Community during Winter 2023—A Cross Sectional Study
Source: Diseases. 2023 Sep 15;11(3):122. doi: 10.3390/diseases11030122 (PMC10529898; doi:10.3390/diseases11030122)
Supplement: Supplementary file 1 [file diseases-11-00122-s001.zip › diseases-2552659-supplementary.pdf]

Supplementary file

Principal of assay

Antibodies specific to virus proteins are coated on the test line region of the nitrocellulose membrane. During testing, antigens of each virus in the specimen react with the antibodies that are coated onto gold nanoparticles. As the sample flows through the test membrane migrates up to react with the antibodies immobilized on the membrane and generate one colored line in the test region. The presence of this colored line indicates a positive result. To serve as a procedural control, a colored line will always appear in the control region if the test has been performed properly. The test result interpreted after 15 minutes.

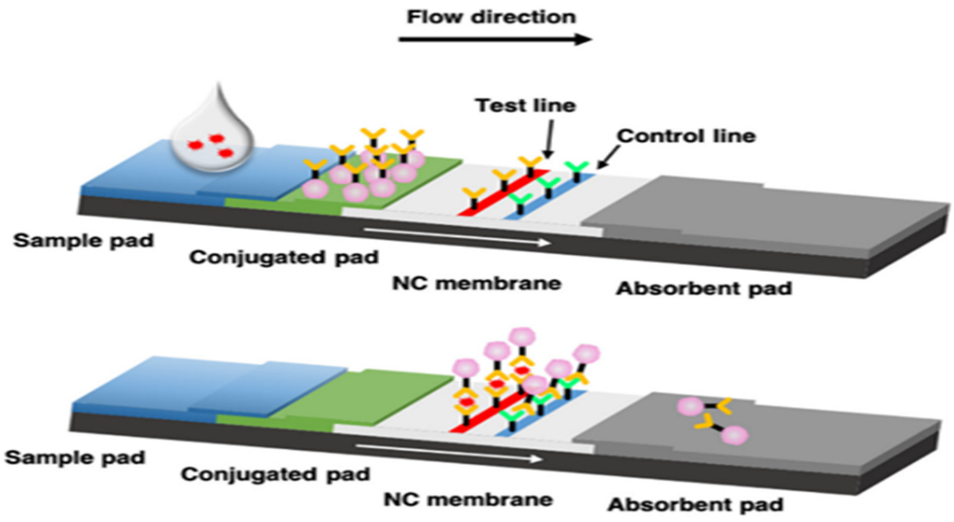

Figure S1. Detection Test Strip

Nasal Mid-Turbinate specimen collection

The patient's head is tilted back 70 degrees. A sterile swab is removed from the pouch. While gently rotating it, the swab is inserted less than one inch (about 2 cm) into patient's nostril (until resistance is met at the turbinate's). The swab is rotated five times against the nasal wall then is slowly removed from the nostril. Using the same swab, the collection procedure is repeated with the second nostril figure 2.

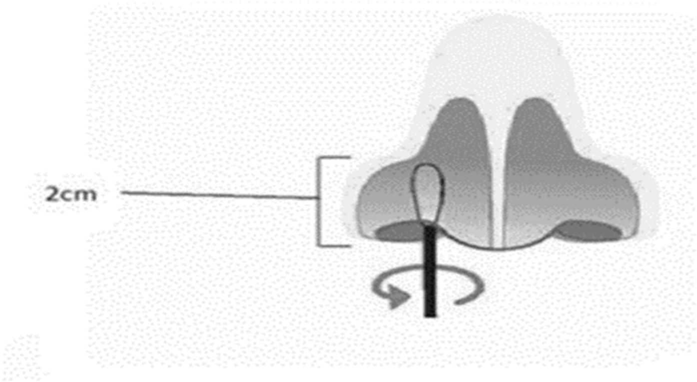

Figure S2. specimen collection

Method Procedure

- After the specimen collection, the swab is placed in the extraction tube, by rotating the swab forcefully against the side of the tube for 1min. Best results are obtained when the specimen is vigorously extracted in the solution.
- The swab is removed, squeezing the sides of the tube to extract as much liquid as possible.
- The swab is discarded.
- The extraction tube is closed with the dropper cup. Two drops are added in the circular window of the cassette for each test.
- After 15 minutes, the test stick can be visually read and interpreted according to the corresponding figure 3.

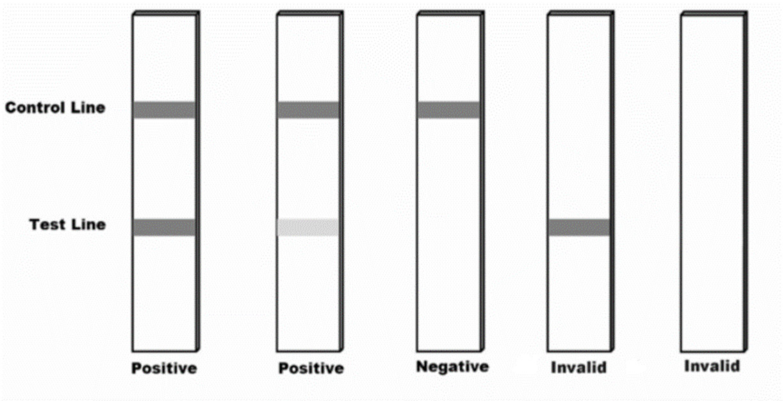

Figure S3. read test
